# Differentiation MicroRNAs Affect Stemness Status of USSCs

**Published:** 2011-10-01

**Authors:** F Jamshidi Adegani, L Langroudi, E Arefian, M Soleimani

**Affiliations:** 1 Department of Molecular Biology and Genetic Engineering, Stem Cell Technology Research Center, Tehran, Iran; 2 Department of Virology, Tarbiat Modares University, Tehran, Iran; 3 Department of Stem Cell Biology, Stem Cell Technology Research Center, Tehran, Iran; 4 Department of Hematology, Tarbiat Modares University, Tehran, Iran

**Keywords:** miRNA, mir-145, Let7g, Unrestricted somatic stem cells, Embryonic stem cell, Pluripotency, Locked nucleic acid

## Abstract

**Background:**

MicroRNAs are endogenous non-coding RNAs with important regulatory and cell fate functions. Many studies have shown that several microRNAs are obviously up-regulated during stem cell differentiation. The question rises here is weather inhibiting differentiation will affect the stemness and self renewal status of stem cells.

**Methods:**

miRCURY ™LNA microRNA inhibitor (anti-miR-145 and anti-let7g) are a sequence-specific and chemically modified oligonucleotide that specifically target and knockdown miR-145 and let7g miRNA molecules. Unrestricted somatic stem cells (USSCs) were isolated from umbilical cord blood and treated with LNAs. The effect of anti-miRNA transfection was assessed by quantitative real-time PCR.

**Results:**

Real-time PCR showed that LNA was efficiently introduced into the cells and reduced miR145 and Let7g expression levels to 40% and 10% in relation to corresponding scramble control, respectively. Gene expression analysis as to self renewal and expansion showed more than 3.5 fold up regulation in Oct4 in cells treated with mir145 inhibition. Similarly a significant up to 2.5 fold up-regulation in Oct4 and cMyc expression was observed in samples treated with anti-let7g.

**Conclusion:**

Suppression in differentiation inducing microRNAs (miR-145 and let7g) can enhance the self renewal and stemness status of USSCs at transcriptional level.

## Introduction

Newly emerged key regulators of gene expression, better known as microRNAs are small non-coding RNAs with about 22 nucleotide length. These tiny elements function by inhibiting translation and protein synthesis. Recent studies have shown the power of microRNAs in cellular processes in all multicellular organisms including development, metabolism and ageing[1] specially in the regulatory circuitries that control self-renewal and pluripotency.[2] It has been shown that several microRNAs increase during differentiation, suggesting a critical role in maintaining cell cycle and self-renewal.

After the first generation of iPS,[3] many methods have been developed but the common, well defined protocol known as the “Yamanaka’s method” is performed by overexpressing a set of specific genes (Oct4, Sox2 cMyc, Klf4) that are highly expressed in ESCs.[3][4] Thomson and coworkers produced iPS cells by transforming human somatic cells into pluripotent stem cells using Oct-3/4, Sox2, Nanog, and a different gene, lin28, with a lentiviral system.[5] It has been suggested that apart from transcription factors and epigenetic modifications, microRNAs can also contribute to cell reprogramming.[6][7]

It is interesting that the aforementioned reprogramming gene lin28 inhibits the biogenesis of the let7 family of microRNAs,[8][9] which has a role in the propagation of breast cancer cells.[10] Therefore, lin28 may actually promote reprogramming by the initiation of differentiation induced by the let7g microRNA. Another miRNAs that have been detected to be of major importance and prevalence among adult cells is mir145 which has also been demonstrated to work against pluripotency, ie. initiating differentiation. It has been shown that both of these elements increase during ESC differentiation.[11]

As mentioned, it has been shown that specific miRNAs regulate mammalian cellular differentiation and developmental patterning in a tissue specific fashion. One of the largest miRNA families is let-7 which indicates such activities.12-20 The well recognized let-7 family is comprised of 12 family members located on 8 different chromosomes.[21][22][23] The sequential expression of let7 RNAs has shown to regulate and synchronize specific stages of development.[24][25] A recent study has shown that lin28 inhibits an early transcript of let7g which is expressed in somatic cells. Let-7g levels have been demonstrated to be regulated by lin28, a protein highly expressed in pluripotent cells, by inhibiting the Dicer-mediated processing of pre-let-7 to mature let-7.[9][26]

It is worth mentioning that the promoters of both let-7 g and lin28 are occupied by the embryonic transcription factors Oct4, Sox2, Nanog, and Tcf3 in mice suggesting that these factors promote the transcription of both primary let-7g and lin28, which then blocks the maturation of let-7 g.[27]

Kosik et al.[11] found that mirR-145 functions to regulate as well as modulate the differentiation progress in hESC differentiation through Oct4/Sox2 pathway. Identification and unique conservation of mir 145 seed sequence in many species (mice,[22]human[28]) and organs such as uterus, ovary, testis, prostate, spleen, muscle and heart[19][29] showed the evolutionary importance and critical impact of this microRNA in cell fate.[30] In line with bioinformatic predictions; targetScan,[31] miRBase[32] and Miranda,[33] it was demonstrated that mir145 represses pluripotency and controls ESC differentiation through interaction with three core pluripotency factors Oct4, Sox2, and Klf4.[11] Up-regulation of miR-145 expression caused a significant diminution of the self-renewal marker SSEA-4 and an increase in multiple differentiation markers associated with all three germ layers.[11]

A new adult stem cell isolated from umbilical cord blood, which has high propagation potential, low immunogenicity and easy isolation, are unrestricted somatic stem cells (USSCs). USSCs are a rare population of intrinsically pluripotent cells in human cord blood.[34][35] These cells do not express CD45, grow adherently, and can be expanded without losing pluripotency. These cells can homogeneously differentiate into osteoblasts, chondroblasts, adipocytes, hematopoietic, and neural cells, including astrocytes and neurons in vitro[35][36] and in vivo, also showed differentiation to mesodermal and endodermal pathways in animal models.[37] Fallahi-Sichani et al.[38] reported that USSCs can be differentiated in vitro into neuron-like cells expressing genes associated with development and/or survival of dopaminergic mesencephalic neurons.

Although several studies have shown the importance of microRNAs in cell fate and their effect on pluripotent genes, there is no study to examine the impact of inhibiting further differentiation in adult stem cells. Working with USSCs, we made a profile of the microRNAs that are expressed in these cells (data not published) which shows that both mir145 and let7g are expressed in these cells. Considering the potential role of miRNAs in cell fate, in the present study, we applied miRCURY LNA™ miR-145 inhibitor (anti-miR-145) and let7g inhibitor (anti-let7g) to knockdown miR-145 and let7g molecules in unrestricted somatic stem cells, and evaluated the effect of these microRNAs on stemness. The anti-miRNA inhibitors are sequence-specific oligonucleotides that specifically target and knockdown miRNA molecules, and have been applied to investigate miRNA functions in several studies.[11][39][40] The results suggested that suppression of miR-145 can induce favourable gene expression in the self renewal pathway but not strong enough to make a considerable difference.

## Materials and Methods

USSC isolation was performed by collecting human umbilical cord blood with informed consent of the mother according to kogler et al. protocol.[35] The mononuclear cell fraction was isolated by Ficoll (Biochrom) gradient separation accompanied with subsequent lysis of RBCs by ammonium chloride. An average portion of 6–7_10(6) cells/ml was plated in T25 culture flasks (Costar). For initial growth of the adherent USSC colonies, two different medias were employed including myelocult medium (Stem Cell Technologies) and low glucose DMEM (Cambrex) with 30% FCS, dexamethasone (10_7 M; Sigma-Aldrich), penicillin (100 U/ml;Grünenthal), streptomycin (0.1 mg/ml; Hefa-pharma), and ultraglutamine (2 mM; Cambrex). After isolation, USSCs were expanded in high DMEM medium, supplemented with 10% FBS.[34][35][38]

USSCs were transfected with 50 nM of miRCURY

LNA™ (Exiqon) targeting each miRNA using lipofectamin 2000® (Invitrogen). Twnty four hours prior to transfection, 2×10(4)cells were plated into 12- well plates to acquire 50% cell density at the time of transfection. The oligomer-lipofectamine™ 2000 complexes were prepared as mentioned in the manufacturer protocol using Opti-MEM as diluents. Samples were harvested at three time points including days 2, 3 and 12 after transfection. To obtain maximum knockdown, two additional booster treatments were applied at day 3 and 5 after the first transfection.

Knocking-down miR-145 and let7g was performed with anti-sense LNA oligomers. miRCURY LNA knockdown probes target sequence for miR-145 (miRCURY knockdown 412385-00), is 5’-GGATTCCTGGGAAAACTGGA-3’, for miR- let7-g (miRCURY knockdown 410026-00) is 5’-ACTGTACAAACTACTACCTC-3’, and for scramble miRNA (miRCURY knockdown, 19900204 scramble-miR) LNA probes as negative control, were purchased from EXIQON, Inc. The same protocol for siRNA transfection was performed to tansfect LNA probes into cells using lipofectamine™ 2000 as described above.

The repression of miRNAs was assessed both 48h and 12 days after the first transfection. miRNA extraction of samples was carried out by our modified total RNA isolation with QIAzol lysis reagent. The RNA concentration was quantified using an Eppendorf AG biophotometer (Eppendorf).

The detection of miRNAs was performed with 1st strand cDNA synthesis kit (Agilent Technologies, Stratagene Products Division) which provides the reagents to elongate miRNAs in a polyadenylation reaction and then reverse transcribe the polyadenylated RNA into QPCR-ready cDNA. The cDNA may then be amplified using the provided universal reverse primer and a unique forward primer that is specific to the miRNA target of interest. Respectively,the primer sequence for miR145 is 5’- GTCCAGTTTTCCCAGGAATCC-3’ and for let7g is 5-TGAGGTAGTAGTTTGTACAGTT-3’. Forward primer of U6–snoRNA (5’- AAATTGGAACGATACAGAGAAG-3’) was used as the endogenous control. The extended protocols are provided online by the manufacturer. Briefly, reverse transcription of 1 μg total RNA by the 1st-strand cDNA synthesis kit (Agilent Technologies) was followed by 45 cycles of realtime PCR using the miRNA QPCR master mix (Agilent Technologies, Stratagene Products Division). miRNA levels were normalized against the snoRNA control.

The polyadenylation reactions are prepared by adding 4 μl of 5× poly A polymerase buffer 1 μl of rATP (10 mM), required appropriate amount of RNA (1 μg ) with RNase-free water to bring to a final volume of 20 μl. Addition of 1 μl of E. coli poly A polymerase to each reaction fallowed by 30 minutes of incubation at 37°C A further 5 minutes at 95°C to terminate adenylation and immediate transfer to ice which is essential at this step.1st-strand cDNA synthesis is the subsequent reaction prepared by adding the following components: 2 μl of 10× affinity script RT buffer, 4 μl of the polyadenylation reaction in the last step, 0.8 μl of dNTP mix (100 mM), 1 μl of RT adaptor primer (10 μM) and 1 μl of affinity script RT/RNase block enzyme mixture. Incubate the reactions at 55°C for 5 minutes, followed by 15 minute incubation at 25°C and subsequent 42°C for 30 minutes to allow reverse transcription of 1st-strand cDNA and finally, 5 minutes at 95°C to terminate the reverse transcription. To screen specific amplification, a no-PAP control cDNA was prepared from a polyadenylation reaction in which the poly A polymerase was omitted. Quantitative Real time PCR reaction was carried out according to manufacturer instructions using a unique forward primer and a universal primer provided in the kit as the reverse primer in the amplification process.

For RT-PCR analysis, total cellular RNA was extracted using QIAzol-reagent (Qiagen). Synthesis of cDNA was carried out with MMuLV reverse transcriptase and random hexamer, according to the manufacturer’s instructions (Fermentas). Primers used for qPCR were shown in supplementary Table 1. The GAPDH transcripts were used as internal control. PCR amplification was performed using Maxima ™SYBR green/fluorescein qPCR Master mix (Fermentas) with a two step procedure of an initial denaturation at 95°C for 10 minutes, followed by cycles circulating 15 seconds of 95°C and 60 seconds of annealing/extension at 60°C. The number of cycles varied between 30 and 40, depending on the abundance of particular mRNA. The primers and product lengths were listed in supplementary Table 1. All reactions were performed in triplicates and normalized to the internal control gene. Changes in microRNA and mRNA expressions were normalized to the relevant internal control, and subsequently calculated using the 2-ΔΔCt method. The rotor gene 6000 detection system (Corbett) was used for quantitative miRNA and mRNA transcript expressions.

**Table 1:** The primers and product lengths (Supplementary).

**Gene**	**Forward**	**Reverse**	**Product size (bp)**
Oct4	CGC CGT ATG AGT TCT GTG	GGT GAT CCT CTT CTG CTT C	284
Sox2	GGA CTG AGA GAA AGA AGA GGA G	GAA AAT CAG GCG AAG AAT AAT	196
cMyc	AGC GAC TCT GAG GAG GAA C	CTG CGT AGT TGT GCT GAT G	183
Lin28	AAA GGA GAC AGG TGC TAC	ATA TGG CTG ATG CTC TGG	107
GAPDH	CTC TCT GCT CCT CCT GTT CG	ACG ACC AAA TCC GTT GAC TC	113

## Results

The transfection efficiency of the LNAs was examined using a negative control (miRCURY knockdown scramble-miR, 199002-04) 24 hours after transfection. The same protocol for transfection of siRNAs using the lipofectamine 2000® reagent was applied. The efficiency was calculated by counting ten fields of view and taking into account the ratio of fluorescent cells (signals omitted from FITC conjugated LNA) to all the cells countable in each view. Working with lipofectamine™ 2000 reagent showed a minimum of 70% transfection ratio. This shows that the majority of USSCs have possessed the LNA for maximum knock down of target miRNA. These cells were then used as negative controls for relative quantification.

**Fig. 1:**
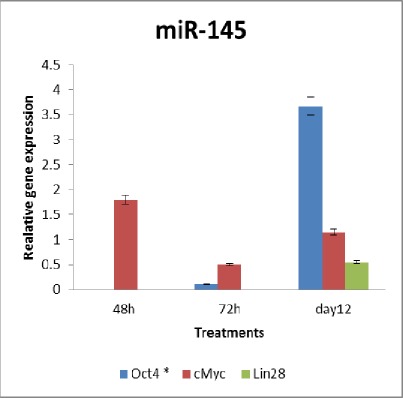
Gene expression after knockdown of mir145.

**Fig. 2:**
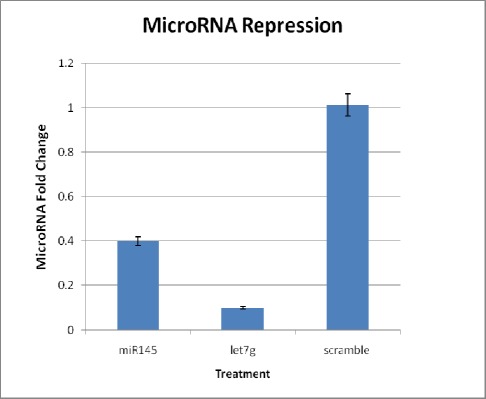
Knockdown of microRNAs by LNA knockdown probes targeting mir145 and let7 g.

Real-time PCR analysis revealed that anti-miR- 145 transfection significantly reduced miR-145 levels to 40% in USSCs compared to negative scramble control (Figure 1), confirming that anti-miR-145 was efficiently introduced into the cells and knocked down miR-145. Also USSCs was transfected with anti- let7 g, and showed that the let7 g levels reduced to 10% and the expression levels persisted until day 12 (Figure 2).

Expression levels of the indicated miR 145 and let7 g with corresponding anti-miR treatments was demonstrated after 12 days of transfections. The miRscramble was the no-target control which had no targets. USSCs were transfected with miRCURY LNA knockdown probes against the named hsa-mir targets and the inhibition was analyzed relative to an internal RNA control, the sno-RNA U6. Each experiment was performed in duplicates, accompanied with a representative control. Statistical significance of the results were assessed using student t-test (*p<0.05).

To assess the effect of treatments with LNA compounds, target genes which our microRNAs regulated were examined with quantitative real time PCR. As mentioned, Oct4, Klf4, cMyc and Sox2 were the direct targets for mir145. Likewise, the target for let7 g was shown to be lin28. After transfection, the cells were harvested and analyzed for gene expression in 3 time points. Samples were collected 48 and 72 hours after transfection accompanied with a final analysis at day 12. To gain maximum knockdown, booster treatments were applied in two time points, days 3 and 5. Samples of each time point were analyzed with corresponding controls.

Expression of mir145 was reduced by 60% (Figure 1) versus corresponding control and it persisted for 12 days after transfection of USSCs with targeted LNA compounds. At 48 and 72 hours after the transfection, levels of target gene expression were compared to the negative scramble control and the final microRNA and target genes were examined 12 days after the first transfection.

An approximately 3.6 fold up regulation in expression of Oct4 was detected in response to downregulation of mir145 (Figure 1). The significant and gradual upregulation of Oct4 initiated 72 hours after the transfection and continued to day 12 where the highest expression was detected.

Also, anti-miR145 treated cells were analyzed for other target genes including cMYc, Sox2 and Klf4 with qRT-PCR analysis. cMyc expression also showed an up-reguation approach in response to the treatment, but the changes were not as significant as Oct4 expressions. It is worth noting that it appears that cMyc expression was altered with each booster treatments and the up-regulation was a direct effect of miR145 repression but the up-regulation did not persist to make a significant difference. qRT-PCR analysis of Sox2 and Klf4 expression did not amplify any products, correlating with controls.

Considering the effect of lin28 in let7 g expression and the indirect effect of the gene in differentiation, we assessed the existence of the gene in anti-mir145 treated cells. The results showed a 45% decrease in lin28 for treated USSCs. Regarding that, after 12 days of treatment the expression level of mir145 had decreased to 60% but the level of let7 g showed an increase during this time (data not shown). At 48 hours post-transfection, expression of target and internal control genes were analyzed by RT-qPCR. Expression of target genes was normalized to GAPDH and is presented as the fold change in expression relative to miR-scramble treatment.

USSCs were transfected with miRCURY LNA knockdown probes against the named hsa-mir targets and the inhibition was analyzed relative to an internal RNA control, the sno-RNA U6. Expression levels were measured using quantitative real time PCR and the miRNA QPCR master mix. Each experiment was performed at least two times, in duplicates, accompanied with a representative control. * Statistically significant alterations in gene expression were compared to the corresponding controls. Statistical significance of the results were assessed using one way Anova (*p<0.05).

**Fig. 3:**
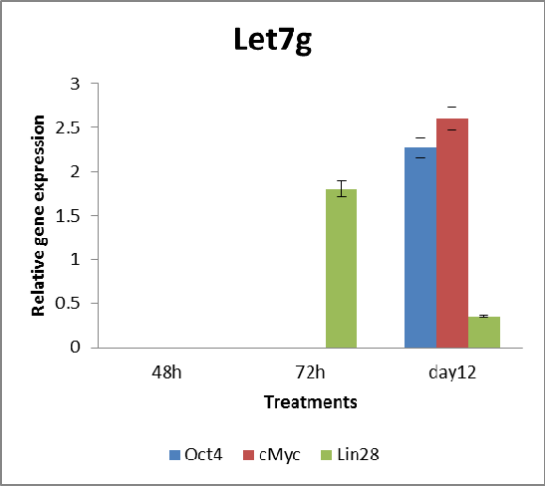
Gene expression changes in let7 g-inhibited USSCs.

Targeting let7 g in USSCs, expression of let7 g was reduced by 90%, versus corresponding controls at day 12 (Figure 3). Although the only known target of let7 g was lin28 gene, we also examined the expression of four other pluripotent markers for any changes induced by let7 g knock down. The direct effect of let7 g inhibition was notably the upregulation of lin28 within 72 h of transfection. The changes detected after induction was the expression of Oct4, cMyc on day 12. With approximately 2.5 fold upregulation in both Oct4 and cMyc, significant up-regulation could only be observed 12 days after transfection. The surprising fact that, regarding the over expression of lin28 after instant knockdown of let7 g, it seems that it could not be maintained until day 12, considering the booster treatments. The expression levels of Sox2 and Klf4 were again not determined due to an absence in amplification. Inhibition of let7 g expression in USSCs by specific sequence specific anti-miR was determined by RTqPCR analysis. Transfection of anti-let7 g and scramble Ccontrol were also carried out using lipofectamine 2000 reagent.

qRT-PCR analysis of let7 g-inhibited USSCs and the corresponding scramble control. The expression levels of Oct4, c-Myc and lin28 were obviously different, where in the case of pluripotent genes Oct4, and cMyc up-regulation relative to the control was observed. The expression levels were normalized to GAPDH gene and are presented as the fold change in expression relative to no treatment. * Statistically significant alterations in gene expression were compared with the corresponding controls. Statistical significance of the results were assessed using one way Anova (*p<0.05).

## Discussion

We were interested in investigating the effect of microRNAs in stem cell fate and self-renewal. The cellular response to crucial microRNAs is of particular importance, especially when the fate of pluripotent genes has shown to be regulated by these minuscule elements.[1][24][41][42] In this study, we looked upon the effect of certain microRNAs on self-renewal factors such as Oct4, Sox2, Klf4, cMyc and lin28 which play an essential role in stem cell maintenance and propagation.[1][8][10][11][43] Treating our target cell, USSCs, with anti-sense oligonucleotids caused a considerable suppression in microRNA expression, where let7 g and mir145 reduced to 10% and 40% relative to scramble control, respectively.

Considering that microRNAs function by reducing the expression level of their targets, we would expect an increase in target gene expression. During treatment of USSCs with anti-miR145, target genes (Oct4, Sox2, Klf4) were examined for changes. At this level, Oct4 expression was the only significant change noticed after treatment. Oct4 showed a significant upregulation throughout the treatment period. This is relevant to previous studies which indicate the role of mir145 in Oct4 expression.[11] But the fact that other targets including Sox2 and Klf4 did not change during the treatment makes a controversial situation.

After 48 hours of transfection, USSCs treated with anti-mir145, showed an up-regulation in cMyc,where as the expression of other defined targets (Oct4, Sox2, Klf4) showed no changes. But examining the results of gene expression 72 hours after transfection revealed that the level of cMyc expression reduced to half, which suggests that in addition to miR145, there are other regulators for cMyc which act to maintain the level of cMyc transcript. In addition, cMyc continued to be regulated despite the reduction observed in miR145 after 12 days of treatment. Although this change in gene expression was not statistically significant, but the pattern produced confirms that adjacent to microRNAs, other regulatory systems are involved.[44][45][46]

Although the insignificant change in cMyc expression was suggested to be contributed to the importance of the regulatory role of cMyc in the cell cycle, and also the oncogenic potential of this factor, [47][48] the unrestrained expression of this gene could result in totally different scenario for stem cell fate.

In the case with anti-let7 g treated USSCs, the results indicate that the target gene, Lin28 is considerably up-regulated after 72 hours of transfection. Although this shows a direct effect of let7 g on lin28,[8][49] but this does not persist until the end of the trial. At day 12, lin28 expression reduces in comparison to relevant controls, producing the same effect observed in the parallel experiments with anti-miR145. As observed in both occasions, it seems that the target genes resist the inhibition induced through anti-sense transfections. Considering that both cMyc and lin28 play a crucial role in the cell cycle, perhaps the opposing act observed to resist the up-regulation of essential factors considered for self-renewal, it is regulated accurately that maintenance in gene expressions are decisive to cell fate.[30] As seen, the controversial fate of a cell is presumed to be “fine tuned” rather act directly in the circuitry involved for preserving the stem cell status. It is interesting that the complex network controlling self-renewal also inhibits factors contributing to uncontrolled cell propagation.[18][50]

Although lin28 up regulation does not persist but the results signify another observation, where the expression of Oct4 and cMyc seems to up regulate to approximately 2.5 fold compared with untreated controls. The transition in gene expression is probably an indirect effect of transient lin28 up-regulation or other affecting sources. As seen with anti-mir145 treated cells, Sox2 and Klf4 expression did not change due to anti-let7 g treatment. Perhaps the elements acting upon these factors are either not targeted by let7 g and the indirect effect observed with cMyc and Oct4 is considered irrelevant.

All together, the indirect and direct effects observed in this studies point to the potentials of microRNAs in stemness of somatic stem cells. Although the act of one microRNA may not be adequate to change the fate of stem cell, but it has the potential to manipulate the accurate regulation of pluripotency network. Stemness is often considered by significant expression of pluripotent genes such as Oct4, Nanog, Sox2, … but to define the exact criteria as to stemness is controversial.[51]

Working towards better understanding of the process controlling the “stemness” of stem cells, it is crucial to select a reliable and high standard source of stem cell for examination. In this study, considering the ease of isolation, low immunogenicity and high propagation potential, made USSCs the desired cells for examination. Taking into account that USSCs are pluripotential stem cells isolated from umbilical cord blood, which was shown to be an abundant resource for a verity of stem cells, and accessible through cord blood banks, the aforementioned criteria make USSCs a valuable candidate for therapeutic purposes. Considering that the source of somatic stem cell is an essential factor in cell based therapies, it is best to work on available resources such as cord blood banks which are expanding by day.
